# Infectious tenosynovitis with bloodstream infection caused by *Erysipelothrix rhusiopathiae*, a case report on an occupational pathogen

**DOI:** 10.1186/s12879-016-2102-1

**Published:** 2017-01-05

**Authors:** Kristine Hofseth, Håvard Dalen, Leif Kibsgaard, Solrun Nebb, Angela Kümmel, Arne Mehl

**Affiliations:** 1 Department of Orthopedic Surgery, Levanger Hospital, Nord-Trøndelag Hospital Trust, Levanger, Norway; 2 Department of Medicine, Levanger Hospital, Nord-Trøndelag Hospital Trust, Levanger, Norway; 3 Cardiac Exercise Research Group, Department of Circulation and Medical Imaging, Norwegian University of Science and Technology (NTNU), Trondheim, Norway; 4 Department of Cardiology, St. Olavs University Hospital, Trondheim, Norway; 5 Department of Laboratory Medicine, Levanger Hospital, Nord-Trøndelag Hospital Trust, Levanger, Norway; 6 Mid-Norway Sepsis Research Group, Norwegian University of Science and Technology, Trondheim, Norway

**Keywords:** *Erysipelothrix rhusiopathiae*, Infectious tenosynovitis, Sepsis, Bloodstream infection, Endocarditis, Surgical treatment, TNF-α inhibitor, Case report

## Abstract

**Background:**

*Erysipelothrix rhusiopathiae* is an established animal pathogen, which may cause infections in humans. It is a gram-positive rod and found in the tonsils or the digestive tracts of animals. The bacterium is occupationally related, as usually only people with frequent animal contacts are infected. We report a case of a patient who was admitted with an infectious tenosynovitis with bloodstream infection due to *E. rhusiopathiae*, and to our knowledge, this is the first report of a tenosynovitis with systemic manifestation associated with this bacterium.

**Case presentation:**

A 52-year old Norwegian man, who worked with transportation of swine cadavers, was admitted to the local hospital with sepsis and unknown focus of infection. A few days earlier he had an injury to the skin of one of his fingers that later proved to be infected with *E. rhusiopathiae*. There were no other causes for his symptoms than the infectious tenosynovitis with systemic manifestation. The infection resolved on treatment with antibiotics and surgery. A transoesophageal echocardiogram was performed to exclude endocarditis, which may be associated with this pathogen.

**Conclusions:**

This case report highlights the importance of clinicians being aware of this bacterium, and we describe risk factors for infection, differences in the clinical manifestations of the disease, challenges with diagnosing the bacterium and adverse effects of immunosuppressive drugs. Recommended treatment is appropriate antibiotic therapy and adequate debridement and surgical drainage of the tendon sheath.

**Electronic supplementary material:**

The online version of this article (doi:10.1186/s12879-016-2102-1) contains supplementary material, which is available to authorized users.

## Background

*Erysipelothrix rhusiopathiae* is described as a non-motile, non-sporulating, non-acid-fast, slender gram-positive rod with capsule, which is easily decolourised. The rod is recovered from the tonsils or the digestive tracts of different animals [1]. Swine is believed to be the major reservoir of *E. rhusiopathiae*, but rodents and birds are also frequently infected. The bacterium also grow and persist in mucoid exterior slime in fish [2]. Humans can be infected from contact with these animals, their secretions, wastes or products, or contaminated organic matter [1].

*E. rhusiopathiae* causes mainly three types of infections in humans. Firstly, a mild cutaneous infection (erysipeloid), described as a local cellulitis, usually on the hands or fingers. The disease is self-limiting within 3–4 weeks without therapy, but if not treated with antibiotics there is a risk of relapse. Secondly, it may cause a diffuse cutaneous infection where the patient experiences that the local lesions spread to other locations of the body and bullous lesions may coexist with systemic symptoms such as fever, malaise, headache, joint- and muscle pain. Some patients also experience polyarthritis. Lastly, *E. rhusiopathiae* may cause septicaemia and endocarditis [1, 2]. Penicillin G is the drug of choice to treat infections caused by this bacterium [2].

Infectious tenosynovitis refers to the infection of a tendon and its synovial sheath. In the setting of tenosynovitis, the space between the inner visceral layer adherent to the tendon and an outer parietal layer may be filled with inflammatory or purulent fluid. Bacteria can enter the tendon sheath by direct inoculation via trauma or spread from infected adjacent soft tissues. It may also spread hematogenously. The most common pathogens are skin flora (i.e., gram-positive cocci such as *Staphylococcus aureus* and streptococci) [3, 4]. Recommended treatment for this type of infection is appropriate antibiotic therapy, adequate debridement and surgical drainage, a period of immobilisation and elevation of the extremity, and early mobilisation. Appropriate tetanus prophylaxis is necessary [4].

Hereby, we describe the successful management of a patient who was admitted with an infectious tenosynovitis with bloodstream infection due to *E. rhusiopathiae*. Only once in published literature there has been described tenosynovitis caused by this bacterium, as a local infection [5]. To our knowledge, this case report shed new light on the pathogenesis of this disease being the first known report of a tenosynovitis with systemic manifestation associated with this bacterium.

## Case presentation

### Admission

A 52-year-old man was admitted to the local hospital because of acute fever and poor general condition. By admittance he was septic with unknown focus of infection. He worked in the transportation business and mainly by transportation of cadavers, most often swine cadavers. He rarely used protective gloves. He had known ankylosing spondylitis since years and was treated with adalimumab injections every 14 days and ibuprofen. He had earlier experienced a reaction to penicillin with symptoms of urticaria. Five days before admission he got a cut on the volar side of the proximal inter phalangeal joint of the fourth finger on the left hand, but he could not remember how he got it. Two days later he had increasing pain in his finger and it swelled, but no pus was seen from the wound. The last 24 h before admission, he experienced fever with chills, increasing pain in the finger, nausea, diarrhoea and fatigue.

On physical examination at admission, his body temperature (after taking antipyretics) was 38.4°C, pulse rate was 93 beats per minute, respiratory rate was 20 per minute, and blood pressure was 147/92 mmHg. By auscultation the lung sounds were normal and there was no cardiac murmur. The abdomen was soft and free from pain by palpation. Examination of the fourth finger on the left hand showed a small, dry wound from the cut, some tenderness along the course of the flexor sheath, slightly flexed finger at rest, erythema and enlargement compared to the other digits.

Laboratory and imaging examinations showed white blood cells (WBC) 18.3 × 10^9^/L, neutrophil granulocytes 16.4 × 10^9^/L, haemoglobin 13.7 g/L, platelets 300 × 10^9^/L, C-reactive protein (CRP) 15 mg/L, lactate dehydrogenase 287 U/L. Blood cultures (three bottles from one venipuncture) were sampled. The chest X-ray was normal and he had a normal urine dipstick test. In conclusion the patient fulfilled 4/4 systemic inflammatory response syndrome (SIRS) criteria and the most plausible explanation was sepsis due to a bacterial tenosynovitis in the fourth finger of the left hand. Based on this the patient was rapidly brought to the operating room.

### Therapeutic intervention

In local anaesthetic ulnar and median nerve block an incision was made to reach the common synovial sheath 2–3 cm proximally to the basis of the fourth finger. Immediately accumulated fluid emptied the synovial sheath and a bacterial sample was collected. In accordance with Norwegian sepsis guidelines in patients allergic to penicillin, the patient was then given clindamycin and gentamicin intravenously. Furthermore, two incisions on the volar side of the proximal and distal interphalangeal joints through the synovial sheath were made and a drain tube was inserted into the sheath from the palm of the hand to the distal interphalangeal joint. The drain tube was connected to Ringer’s acetate that flushed the tendon sheath until the next day.

### Recovery and discharge

The day after the patient was feeling much better. He had no fever and did not appear septic. Laboratory examinations showed: WBC 19.7 × 10^9^/L; CRP 73 mg/L. Three out of three blood cultures bottles were positive with gram-positive rods, and gram-positive cocci in clusters, probably staphylococci, were found in the sample from the synovial sheath. The patient was referred for transthoracic echocardiography to exclude endocarditis and the echocardiogram showed morphologically normal valves and chambers with no signs of vegetations. After 4 days of intravenous treatment with clindamycin 600 mg × 3/day and gentamicin 320 mg × 1/day, the patient was discharged in well-being. CRP had fallen to 11 mg/L and WBC to 9.3 × 10^9^/L. He continued with clindamycin orally 300 mg × 3/day for 1 week. He was recommended to avoid the upcoming injection of adalimumab at discharge.

Five days after discharge, a transoesophageal echocardiogram was performed with no signs of valvular or endocardial manifestation of the disease. He was recommended to see his general practitioner for a clinical follow-up and removal of the stitches 1 week after discharge. At an outpatient control after three months and when we contacted him ten months after discharge, the patient reported no relapse of the infection and no persistent sequela.

### Verifying the rod

**Fig. 1:**
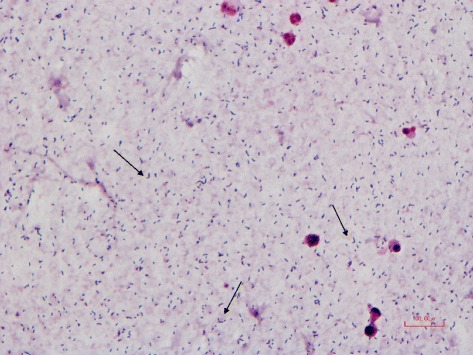
Gram stain smear of *E. rhusiopathiae* from a blood culture bottle. The *arrows* point toward Gram positive rods with V-formation, a characteristic way the bacteria group together

A blood culture set including two aerobic bottles and one anaerobic bottle was sampled immediately at admission. Within the first 24 h of incubation in 35°C, all three bottles turned positive. The gram stain showed gram-positive rods (Fig. 1). Due to characteristic V-formation when visualised in the microscope, they could easily be mistaken for *Corynebacterium sp*. Because of this we did an initial susceptibility test, directly from positive bottles, based on antibiotics used on *Corynebacterium sp.* according to Nordic Committee on Antimicrobial Susceptibility Testing (NordicAST) guidelines with disc diffusion method [6]. The isolate was susceptible to penicillin, clindamycin, ciprofloxacin, linezolid and doxycycline and was resistant to gentamicin and rifampicin.

After six to eight hours there were signs of growth on agar plates, but not enough for further examination. After 24 h of incubation, there was growth on both aerobic and anaerobic agar plates. Identification of the isolate was achieved by automatically testing with BD Phoenix 100. It was identified as *E. rhusiopathiae* with 99% confidence value. Confirmation of the result by the Maldi-tof system was done at the regional university reference laboratory. Due to the patient’s history, the isolation of this bacterium was expected.

A second susceptibility test was done from cultured bottles to determine the minimum inhibitory concentration value of the antibiotics (MIC), using NordicAST guidelines on non-species related breakpoints. MIC-testing showed the isolate was susceptible to penicillins, cephalosporins, ciprofloxacin, clindamycin, erythromycin and imipenem. The isolate was resistant to gentamicin, trimethoprim-sulfa and vancomycin.

During surgery two samples from the synovial sheath were collected and these were also cultured. One sample was sterile, *Staphylococcus aureus* alone was found in the other. The isolate was, among others, susceptible to clindamycin and gentamicin. This bacterium was not detected in the blood cultures.

## Discussion

We have described a rare invasive *E. rhusiopathiae* infection and several lessons can be learned from this case.

Swine are the most important animal reservoir of *E. rhusiopathiae*, which is shed by infected animals in faeces, urine, saliva and nasal secretions [1]. Our patient worked with transportation of swine cadavers, and thus, he had an occupational risk for such infections. We know that 20–40% of healthy swine harbour this pathogen in their lymphoid tissue of the alimentary tract, particularly in the tonsils [1]. Infections caused by *E. rhusiopathiae* are strongly related to the occupation of the patient and almost 90% of patients with endocarditis due to this pathogen have an occupational risk of infection [7].

Most often infection with *E. rhusiopathiae* is initiated either by an injury to the skin with infective material or by contamination of a previous injury. There have also been a few documented cases of penetration through the skin by this bacterium [1]. Our patient rarely used protective gloves, and he had a cut in one of his fingers a few days before admission. We believe that contamination of this injury was the cause of the infection. Due to changes in technology in the animal industry, it has been suggested that the frequency of human infection by this pathogen is decreasing [2]. However, in some environments, exposure to *E. rhusiopathiae* is maintained, and the threat of infections is present. Preventive initiatives include good hygiene with frequent hand washing, use of protective gloves and cleansing of wounds. Removal or regular disinfection of contaminated sources is important to limit the spread of the bacterium as well [1].

Our patient was diagnosed with ankylosing spondylitis since years and was treated with adalimumab, a tumour necrosis factor-alpha (TNF-α) inhibitor. TNF-α is an important component of the immune response to a variety of infections. Thus, the use of such medication has been associated with an increased risk of serious infections [8], including infective tenosynovitis [4]. As the American College of Rheumatology recommends not to administer TNF-α inhibitors to patients with active bacterial infections [9], our patient was instructed not to take the subsequent dose of adalimumab. Depression of the immune system predisposes to the development of systemic infections with among other *E. rhusiopathiae*, and thus, the risk of systemic infections, as endocarditis, is also increased [7].

Reports show that systemic infection with *E. rhusiopathiae* occur in less than 1% of cases, but in 90% of these there is an association with endocarditis [1, 7]. The first case of endocarditis with *E. rhusiopathiae* was reported in 1912, but since then no more than 80 cases of endocarditis caused by *E. rhusiopathiae* have been reported [7]. This cause of endocarditis is therefore extremely rare [7, 10]. However, endocarditis caused by *E. rhusiopathiae* has a high case fatality rate of approximately 40% [10, 11]. Due to these reasons the echocardiographic examinations included a transoesophageal approach as this is more sensitive to reveal endocardial manifestations, and an echocardiographic examination was repeated after 6 days. *E. rhusiopathiae* is reported to be susceptible to penicillin, cephalosporin, imipenem, clindamycin, and fluoroquinolones, but it is often non-susceptible to macrolides and chloramphenicol, and resistant to sulphonamides, vancomycin and aminoglycosides [10]. The first-line empiric therapy for a presumed endocarditis in patients allergic to penicillin include vancomycin and aminoglycosides, which have no effect against *E. rhusiopathiae* [2, 7]. It is therefore important to reveal the correct diagnosis to avoid delayed administration of appropriate drugs. The recommended treatment in patients with endocarditis due to this bacterium is penicillin G given intravenously for a total duration of 4–6 weeks [2]. Alternatively, patients with an *E. rhusiopathiae* infection and coexisting allergy to penicillin can be treated with cephalosporin or fluoroquinolone [2, 12].

Due to sepsis and presumed allergy to penicillin, the patient was treated with an aminoglycoside, to which the bacterium was resistant, and clindamycin. Clindamycin may be bactericide in high concentrations [12]. Our patient responded well to the treatment and did not fill the SIRS-criteria the day after surgery. Thus, we decided to continue with clindamycin orally after discharge for one week. The patient received antibiotic therapy for 12 days in total. The optimal duration of antibiotic therapy for *E. rhusiopathiae* bloodstream infection is unknown and has not been evaluated in clinical trials. Thus, the duration of antibiotic therapy in patients with bacteremia, but without endocarditis, must be based on the clinical response to the treatment provided and the patient’s underlying health condition.

The diagnosis of *E. rhusiopathiae* can be challenging. In this case the blood cultures revealed *E. rhusiopathiae*, but in the sample from the tendon sheath only *S. aureus* was found. This may be caused by the slow growth of the bacterium and the small sizes of its colonies. *E. rhusiopathiae* can be overgrown with secondary pathogens such as *S. aureus* and *S. pyogenes*. We believe that this is what happened with the cultured samples from the tendon sheath. The diagnosis of this pathogen can therefore be challenging if the bacteriologists are not aware of the clinical suspicion of potential contamination from animal contact [1]. Thus, infections with *E. rhusiopathiae* may be under-diagnosed.

According to our recommendations, two blood culture sets from two separate venipunctures should be drawn. In this case, probably because of time constraints, only one blood culture set including two aerobic bottles and one anaerobic bottle was sampled. We have still judged this case to be a real bloodstream infection, as it is unlikely that *E. rhusiopathiae* should occur as a skin contaminant at the venipuncture site.

## Conclusion

This case report shed new light on the pathogenesis of infections with *E. rhusiopathiae* being the first known report of a tenosynovitis with bloodstream infection associated with this pathogen. Recommended treatment for this type of infection is appropriate antibiotic therapy and adequate debridement and surgical drainage. Due to the slow growth of the bacterium, the small sizes of its colonies, and the risk that it can be overgrown with secondary pathogens, the diagnosis of this pathogen is challenging if bacteriologists are not aware of the clinical suspicion of potential contamination from animal contact. This shows the importance of both comprehensive medical history and accurate and detailed information accompanying samples to the microbiology laboratory. Even though *E. rhusiopathiae* is a rare cause of endocarditis or other systemic infections, endocarditis is associated with high case fatality rate. *E. rhusiopathiae* is reported to be resistant to vancomycin and aminoglycosides, which represent the first-line empiric therapy for a presumed endocarditis in patients allergic to penicillin. This highlights the importance of revealing the correct diagnosis to avoid delayed administration of appropriate drugs.

## Additional files

Additional file 1: Gram stain smear from agar plate. Description of data: Gram stain smear of *E. rhusiopathiae* from growth on agar plate. The arrows point toward typical V-shaped bacteria. (PDF 531 kb)
Additional file 2: Timeline. Description of data: Timeline showing patient history and clinical findings, diagnostic evaluation and treatment in chronological order. (PDF 247 kb)

## Abbreviations

CRP: C-reactive protein
MIC: Minimum inhibitory concentration
NordicAST: Nordic committee on antimicrobial susceptibility testing
TNF-α inhibitors: Tumour necrosis factor-alpha inhibitors
WBC: White blood cell
