# Supplementary figures and images for: Infectious tenosynovitis with bloodstream infection caused by Erysipelothrix rhusiopathiae, a case report on an occupational pathogen
Source: BMC Infect Dis. 2017 Jan 5;17:12. doi: 10.1186/s12879-016-2102-1 (PMC5217415; doi:10.1186/s12879-016-2102-1)

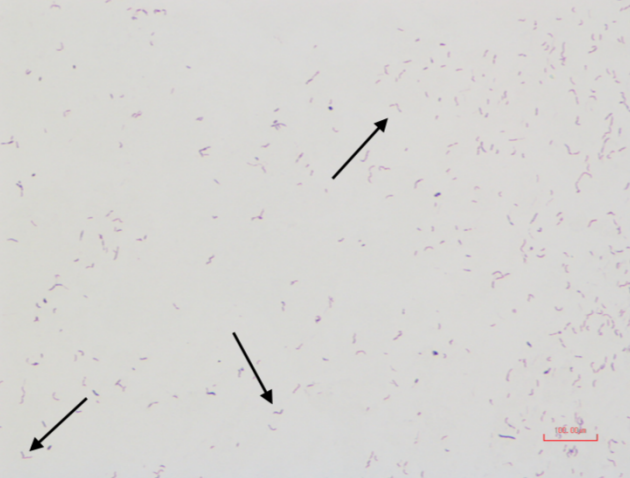

Supplement: Additional file 1: — Gram stain smear from agar plate. Description of data: Gram stain smear of E. rhusiopathiae from growth on agar plate. The arrows point toward typical V-shaped bacteria. (PDF 531 kb) [file 12879_2016_2102_MOESM1_ESM.pdf]

# TIMELINE

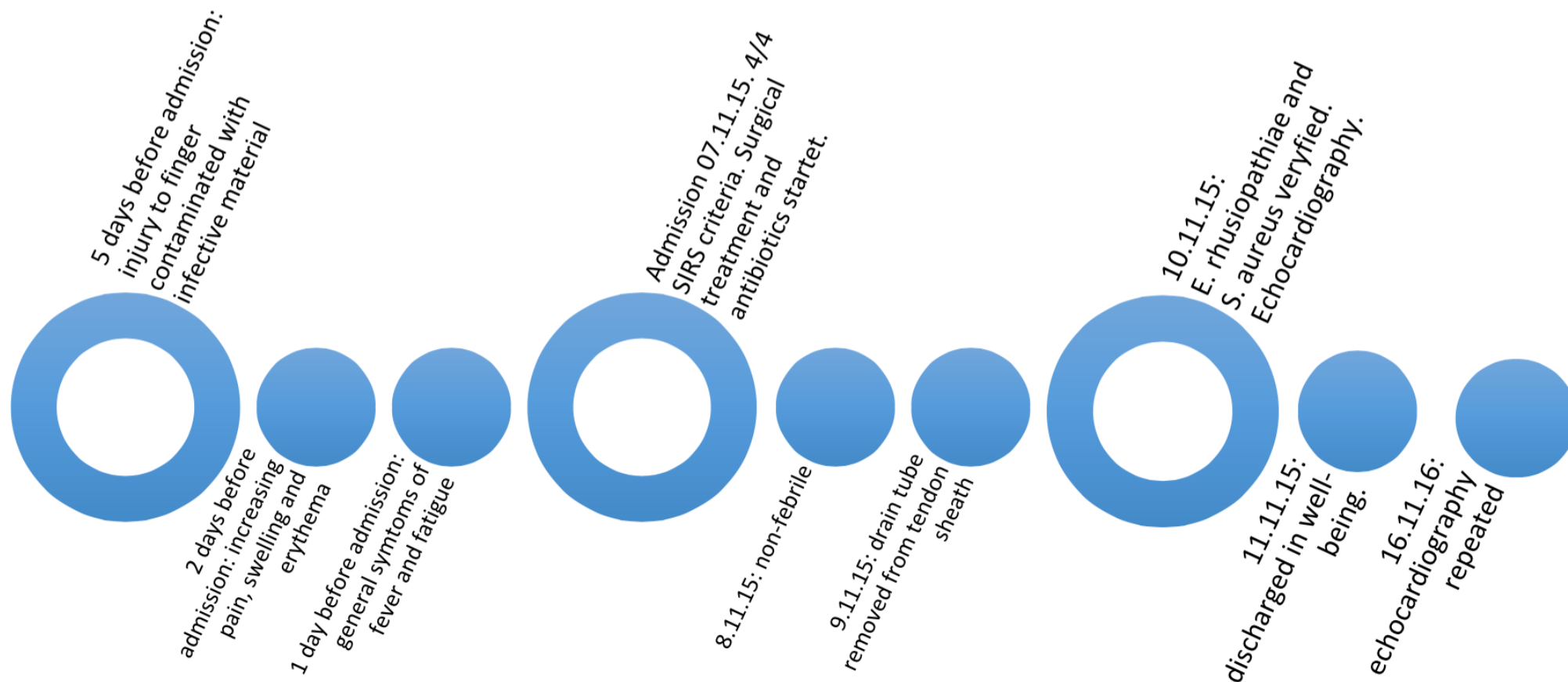

Supplement: Additional file 2: — Timeline. Description of data: Timeline showing patient history and clinical findings, diagnostic evaluation and treatment in chronological order. (PDF 247 kb) [file 12879_2016_2102_MOESM2_ESM.pdf]
